# Mechanical and Material Tendon Properties in Patients With Proximal Patellar Tendinopathy

**DOI:** 10.3389/fphys.2020.00704

**Published:** 2020-06-24

**Authors:** Hans-Peter Wiesinger, Olivier R. Seynnes, Alexander Kösters, Erich Müller, Florian Rieder

**Affiliations:** ^1^Department of Sport and Exercise Science, University of Salzburg, Salzburg, Austria; ^2^Department of Physical Performance, Norwegian School of Sport Sciences, Oslo, Norway; ^3^Institute of Physical Medicine and Rehabilitation, Paracelsus Medical University, Salzburg, Austria

**Keywords:** patellar tendinopathy, VISA-P, tendon viscoelastic properties, tissue function, tissue integrity

## Abstract

**Introduction:**

The effect of chronic patellar tendinopathy on tissue function and integrity is currently unclear and underinvestigated. The aim of this cohort comparison was to examine morphological, material, and mechanical properties of the patellar tendon and to extend earlier findings by measuring the ability to store and return elastic energy in symptomatic tendons.

**Methods:**

Seventeen patients with chronic (>3 months, VISA-P < 80), inferior pole patellar tendinopathy (24 ± 4 years; male = 12, female = 5) were carefully matched to controls (25 ± 3 years) for training status, pattern, and history of loading of the patellar tendon. Individual knee extension force, patellar tendon stiffness, stress, strain, Young’s modulus, hysteresis, and energy storage capacity, were obtained with combined dynamometry, ultrasonography, magnetic resonance imaging, and electromyography.

**Results:**

Anthropometric parameters did not differ between groups. VISA-P scores ranged from 28 to 78 points, and symptoms had lasted from 10 to 120 months before testing. Tendon proximal cross-sectional area was 61% larger in the patellar tendinopathy group than in the control group. There were no differences between groups in maximal voluntary isometric knee extension torque (*p* = 0.216; *d* < −0.31) nor in tensile tendon force produced during isometric ramp contractions (*p* = 0.185; *d* < −0.34). Similarly, tendon strain (*p* = 0.634; *d* < 0.12), hysteresis (*p* = 0.461; *d* < 0.18), and strain energy storage (*p* = 0.656; *d* < 0.36) did not differ between groups. However, patellar tendon stiffness (−19%; *p* = 0.007; *d* < −0.74), stress (−27%; *p*< 0.002; *d* < −0.90) and Young’s modulus (−32%; *p* = 0.001; *d* < −0.94) were significantly lower in tendinopathic patients compared to healthy controls.

**Discussion:**

In this study, we observed lower stiffness in affected tendons. However, despite the substantial structural and histological changes occurring with tendinopathy, the tendon capacity to store and dissipate energy did not differ significantly.

## Introduction

Tendinopathy is a multifactorial tissue disorder (Riley, 2005) responsible for almost every second sport- or occupational-related injury (Jung et al., 2009) and impedes physical function and performance (Maffulli et al., 2003; Lian et al., 2005; Malliaras and Cook, 2006). The precise pathogenesis remains vague, but the disease is often described as overload injury, caused by a mismatch between the functional demand and the adaptational rate of tendinous tissue (Riley, 2004; Magnusson et al., 2010). Degenerated tissue is characterized by essential alterations in the extra-cellular matrix content, including a proliferation of hydrophilic macromolecules (e.g., proteoglycans, glycosaminoglycans; Riley, 2005), proportional changes of type I toward type III collagen (Maffulli et al., 2000), increased crosslinking concentration (Kongsgaard et al., 2009) or lower fibril density (Kongsgaard et al., 2010). Despite some of these changes in tissue composition being able to affect tendon viscoelastic properties, the global effect of tendinopathy on the mechanical properties of the patellar tendon remains elusive (Obst et al., 2018).

Tendons play an essential role by enabling the utilization of elastic energy toward efficient muscle power output and buffering rapid stretch of muscle fibers (Roberts and Azizi, 2011). Muscular function and integrity are thus closely linked to tendon mechanical properties or change thereof. Tendon mechanical properties and their change are thus of paramount importance for muscular function and integrity. While growing evidence suggests that tendinopathic Achilles tendons present a lower stiffness than healthy controls, the studies on patellar tendon have thus far been discordant. *In vivo* measures on tendinopathic patellar tendons currently indicate either a deficit (−20%, Helland et al., 2013) or did not find evidence for differences in tendon stiffness (Kongsgaard et al., 2010; Couppé et al., 2013; Lee et al., 2017) when comparing affected and asymptomatic, healthy sides. Likewise, reports indicate that patellar tendon strain and Young’s modulus are either lower, higher, or similar in affected patellar tendons (Obst et al., 2018).

These inconsistent findings have been ascribed to differences in the severity of the disease, and to the challenges to assess tendon mechanical properties *in vivo* (Lee et al., 2017). Given the few studies (*n* = 4, to date) on this topic, additional research is required to elucidate whether tendinopathy alters the stiffness of the patellar tendon, as suggested by alterations in extracellular matrix composition and by certain studies on patellar (Helland et al., 2013) and Achilles tendons (Wang et al., 2012; Chimenti et al., 2014; Chang and Kulig, 2015) or whether this condition has no effect on the mechanical properties of the patellar tendon (Kongsgaard et al., 2010; Couppé et al., 2013; Lee et al., 2017).

Furthermore, although tensile stiffness has received all the attention of studies assessing the impact of tendinopathy on tendon mechanical properties, there is to date no information regarding tendon elastic hysteresis. The energy dissipation (∼9% in healthy tendons; Bennett et al., 1986) occurring during recoil affects the amount of energy recovered from tendon stretch and, importantly, may be linked to the tissue susceptibility to fatigue damage (Maganaris et al., 2008; Farris et al., 2011; Lichtwark et al., 2013; Herod and Veres, 2017). The current lack of information about tendon hysteresis seems mainly due to the methodological difficulty to test this parameter *in vivo* (Finni et al., 2013). However, over the past few years, some research groups were able to propose reliable methods to measure tendon hysteresis *in vivo* (Kubo, 2004; Peltonen et al., 2012, 2013; Stenroth et al., 2012; Fouré et al., 2013; Wiesinger et al., 2017). Some of these studies indicated that tendon hysteresis levels are related to loading patterns (Reeves et al., 2003; Kubo, 2004; Fouré et al., 2010) in a way that could not be predicted from other biomechanical parameters (Wiesinger et al., 2017). Hence, despite the known plasticity of tendon hysteresis with loading conditions and, possibly, with changes in tendon composition, there is currently an unmet clinical need to characterize the influence of tendinopathy on this parameter.

In this study, we propose to conduct an exhaustive assessment of patellar tendon properties in tendinopathic patients and in matched controls by including for the first time tendon hysteresis measurements. A secondary aim was to extend the body of literature about stiffness of tendinopathic tendons.

We predicted that tendinopathic patellar tendons to exhibit greater cross-sectional area (CSA) of the affected region, but a decreased elastic modulus when compared to asymptomatic, healthy control tendons. Based on the limited evidence, we hypothesized that the chronic patellar tendinopathy of the inferior pole would not meaningfully effect tensile tissue strain and stiffness. Given the complexity of histological changes associated with tendinopathy and in the absence of previous data to specify this hypothesis, the purpose of this exploratory study was also to determine whether hysteresis would be lower or higher with this disorder.

## Materials and Methods

### Subjects

Seventeen patients with chronic (>3 months; Leadbetter, 1992) patellar tendinopathy (24 ± 4 years, male = 12; female 5) and 17 strictly matched healthy controls (25 ± 3 years, male = 12; female 5) volunteered to participate in this cohort comparison study (Supplementary Figure S1). Screening of eligibility was confirmed by a specialist for Physical Medicine and Rehabilitation (J.H) at the Institute of Physical Medicine and Rehabilitation of the Paracelsus Medical University Salzburg. Examination of tendinopathy included persistent but aggravated pain during or after weight-bearing activity, impaired functional performance (VISA-P ≤ 80, Visentini et al., 1998; Zwerver et al., 2011) and palpation tenderness around the affected tissue region. Other or additional knee joint injuries were largely excluded using a comprehensive manual clinical examination (e.g., pivot shift test, Steinmann II test, Lachman test). Longitudinally, ultrasonography and magnetic resonance images verified anterior-posterior tendon thickening of at least 1 mm compared with mid-tendon level (Couppé et al., 2013) irregular tendon structure, hypoechoic area and several cases of neovascularization (*n* = 13, male = 10; female 3) and/or non-tendon-like phenotypes, including bone formation or heterotopic ossification (*n* = 7, male = 6; female 1) at the proximal tendon region only (Black et al., 2004). The present protocol focused on proximal patellar tendinopathy to allow for interstudy comparisons (Kongsgaard et al., 2010; Couppé et al., 2013; Helland et al., 2013; Lee et al., 2017). Non-symptomatic tendons were free of any of these signs of degeneration, and the pain-free group reported no previous history of any known tendon pain. Further exclusion criteria included self-reports of neuromuscular, cardiovascular or respiratory disorders, diabetes, disclosure of anabolic drug abuse, use of oral anticoagulants, any knee surgery, or injections in or along the patellar tendon in the preceding 12 months, Osgood-Schlatter disease or other knee pathologies that potentially impede maximal muscle contraction. Besides, subjects with oral confirmation of claustrophobia or incorporated metallic foreign implants were excluded based on magnetic resonance imaging (MRI). Recruitments were consecutive via local newspaper advertisements, social media, and verbal contacts between March 2017 and August 2019. Purposes, benefits, and risks of testing procedures were given before obtaining written informed consent. The study is part of an ongoing clinical project (German register for clinical trials DRKS00011338) and was approved by the Local Research Ethics Committee (415-E/2012/11-2016).

### Experimental Design

Data of each individual were collected within 2 weeks by the same experienced investigators (H-P.W, F.R) at the Department of Sport and Exercise Science. In patients suffering from chronic patellar tendinopathy, lower leg strength and tendon properties were estimated in the leg of the pathology, or that leg of the most severely affected tissue disorder. In the healthy matched case-control subject, leg selection was based on leg dominance (Büsch et al., 2009) of the paired subject with tendinopathy. All subjects confirmed a 24 h absence of vigorous activities (Shalabi et al., 2004; Boesen et al., 2006) received detailed instructions, contemporaneous visual feedback, and conducted at least two exercise-specific familiarization trials prior to each testing. Investigators provided strong verbal encouragement throughout attempts with resting periods lasting for one minute between tests and half a minute between trials of the same test. Questionnaires were answered without investigator assistance, and all offline analyses were executed in a subject-blinded fashion.

### Patellar Tendon Morphology

Patellar tendon resting length and regional tendon CSA were assessed from sagittal and axial plan magnetic resonance imaging, respectively (3T-Achieva, Philips Healthcare, Eindhoven, Netherlands; lower extremity coil and the following parameters: TR/TE 682/20ms, FOV 100, matrix 528 × 528, slice thickness 3.0 mm, space 3.3 mm), and analyzed offline using an image-processing program (ImageJ, Rasband, W.S., National Institutes of Health, Bethesda, MD, United States)^1^. Tissue length was defined as the distance between the tibial insertion and the apex of the patella. Mean patellar tendon CSA was obtained as the average of three separate measures performed at the proximal (pCSA) and distal (dCSA) insertion and at tendon mid-length (mCSA). Analyses were conducted by the same orthopaedist (YS). The reliability of MRI-based measurements of patellar tendon CSA has been reported in previous publications (Seynnes et al., 2009; Stenroth et al., 2019).

### Maximal Voluntary Contraction

Lower leg strength and tendon properties measurements were preceded by a warm-up routine consisting of 10-min of cycling at 1.5 W⋅kg^–1^ at a cadence of ∼70 rpm on a stationary ergometer (Heinz Kettler GmbH and Co. KG, Ense-Parsit, Germany). The rotational center of the isokinetic dynamometer arm (IsoMed 2000 D&R Ferstl GmbH, Hemau, Germany) was carefully aligned with the rotation axis of the knee joint. Subjects sliding was minimized by means of firm securing with adjustable shoulder pads, hip and footplate straps. Hip and knee joint angles were set to 75° and 90° (0° corresponding to full extension) throughout all test procedures (Dirnberger et al., 2012).

Knee extensor and flexor muscle torques were obtained from two maximal isometric contractions (MIVC), with a gradual but continuous ∼2-s build-up period and a 3-s plateau. The trial yielding the highest peak torque was retained for analysis. Three additional MIVCs were performed at a rate of 50 Nm⋅s^–1^ with visual feedback to test patellar tendon properties (see below). Tensile tendon force was calculated offline as the difference between the net extension torque and the torque produced by antagonist’s muscles, divided by the patellar tendon moment arm, which was based on a function of upper segment leg length (Visser et al., 1990). Antagonist muscle torque was estimated from surface electromyographic (sEMG) activity, by assuming linearity between sEMG and torque produced during knee flexion and extension (Magnusson et al., 2001). To this end, surface electrodes (Ag/AgCL; 120 dB, Input impedance: 1200 GOhm; 10 mm diameter, 22 mm spacing, Biovision, Wehrheim, Germany) were apposed on the biceps femoris and semitendinosus muscles. Raw sEMG signal was filtered offline using a second-order Butterworth filter with a cut off frequency of 10, and 300 Hz. Maximal sEMG amplitude was quantified when the hamstrings acted as agonists as the root mean square of the signal over a 0.5-s period around the peak torque of knee flexion (Wiesinger et al., 2016). This technique has shown high interday reliability (Reeves et al., 2004).

### Patellar Tendon Properties

Patellar tendon stiffness, stress, strain, and Young’s modulus were obtained from isometric ramp contractions up to the maximal level of voluntary exertion. Hysteresis and strain energy were acquired from symmetrical loading and unloading ramps with a fixed peak load of 80% of individual MIVC torque levels (MIVC_80%_). The targeted and produced torque were displayed on visual feedback, and several practice trials were performed for familiarization and tendon preconditioning (Maganaris, 2003) before each test. The reliability of these techniques has been demonstrated previously (Kösters et al., 2014; Wiesinger et al., 2016, 2017).

Tendinous tissue elongation was captured at 29 Hz by placing an ultrasound probe (linear array transducer 5 cm, LA523, 10–15 MHz transducer, MyLab25, Esaote, Genoa, Italy) sagittal over the patellar tendon. Scans were then analyzed offline by an experienced investigator (H-P.W) as the displacement between the patella apex and the tibial anterosuperior aspect using a semi-automatic video analysis software (Tracker 4.87, physlets.org/tracker/). Dynamometer signals and sEMG data were collected at a sampling rate of 2000 Hz, and all data records were synchronized from sending an electrical pulse grabbed simultaneously by the dynamometer and ultrasound system (Matlab, version R2017b; The MathWorks Inc., Natick, MA, United States). Force-deformation and stress-strain slopes were fitted using a constrained least-square function (Wiesinger et al., 2017). Patellar tendon stiffness (Δforce/Δdeformation) and Young’s modulus (Δstress/Δstrain) were calculated from the highest individual and common 10% force interval or stress interval, respectively. Stress (tensile force/CSA) and strain (Δtendon length/tendon length at rest) values reflect the highest tissue force or strain. Hysteresis values were determined as the percent difference between the areas under the ascending and descending phases of the stress-strain curves obtained during MIVC_80%_ trials. Strain energy was calculated as the area under the ascending stress-strain loading curves during the same tests. Each test condition consisted of a minimum of five trials, but load-deformation data were only retained if the torque did not deviate from individual MIVC by more than 5%, and the curve-fitting coefficient of determination R^2^ exceeded 0.90. Where possible, three trials were averaged, however, in some cases only two trials achieved the above requirements.

### Statistics

Statistical analyses were performed using IBM SPSS Statistics V.26.0 (SPSS Inc., Chicago, IL, United States). Two-tailed paired-samples *t*-tests were computed to test for differences between patients and healthy controls in all investigated parameters. The normality of the standardized residuals was analyzed conducting a Shapiro-Wilk test, and the Wilcoxon signed-rank test was applied in case of a non-normal residual distribution. The confidence interval for the mean difference was obtained by using a 95% bootstrap method with bias-corrected and accelerated (BCa). The effect size (*d*) was defined as small for *d*> 0.2, medium for *d*> 0.5, and large for *d*> 0.8 (Cohen, 1988). Pearson correlation coefficients were used to examine the relationship between the differences in the variables of the tendinopathic individuals and matched controls to the severity of the symptoms and functional limitations (VISA-P score). Figures were created using the GraphPad Prism 8.4.2 (GraphPad Software Inc., La Jolla, United States). Unless otherwise stated, results were expressed as mean ± standard deviation (SD). The level of significance was set a *p* = 0.05.

## Results

### Subject Characteristics

Since athletic activity background and volume were used as matching criteria, all pairs of case and control practiced the same sport, at the same national or international level, for a similar number of years and according to comparable weekly volumes. Patients and controls had thus loaded their patellar tendon according to comparable functional requirements. Athletic activity included football (*n* = 12), volleyball (*n* = 8), team handball (*n* = 4), skiing (*n* = 4), high level of long-distance running (*n* = 2), American football (*n* = 2), and recreational activities (*n* = 2). In line with activity background and volume, anthropometric parameters did not differ between groups. VISA-P and VAS scores ranged from 28 to 78 and 3.0 to 9.5 points, respectively, and symptoms had lasted from 10 to 120 months before the examination (Table 1). The symptom time duration was not related to percent differences of matched pairs in tendon proximal CSA (*r*^2^ = 0.12, *p* = 0.171), stiffness measured to individual (*r*^2^ = 0.01, *p* = 0.690) or highest common force levels (*r*^2^ = 0.04, *p* = 0.452), Young’s modulus (*r*^2^<−0.01, *p* = 0.647), hysteresis (*r*^2^ = 0.05, *p* = 0.368), or VISA-P scores (*r*^2^<−0.03, *p* = 0.527).

**TABLE 1:** Anthropometric characteristics, training status, and degree of patellar tendon symptoms.

	Tendinopathy	Controls	*t*-Value_(16)_	Difference BCa 95% CI	*P*-Value	*d*-Value
Age (years)	23.7 ± 1.00	25.0 ± 0.66	1.64	[−0.17, 2.45]	0.119	0.40
Body mass (kg)	76.9 ± 3.3	74.9 ± 3.3	−1.99	[−4.65, 0.18]	0.063	0.44
Body height (cm)	179.3 ± 2.10	178.9 ± 2.41	−1.89	[−4.03, 3.84]	0.925	0.05
BMI (kg⋅m^–2^)	23.2 ± 0.69	23.8 ± 0.80	−0.10	[−1.56, 0.17]	0.076	0.34
Training experience (years)^#^	8.9 ± 1.27	9.6 ± 1.57	1.83	[0.32, 3.86]	0.091	0.44
Training (hours/week)^#^	7.4 ± 1.00	5.6 ± 0.82	−1.17	[−2.38, 0.38]	0.265	0.28
Symptom duration (months)	38.3 ± 8.18					
VISA-P score	56.6 ± 3.49					
VAS	7.1 ± 0.40					

*Values are expressed as mean ± standard error of the mean. BMI, body mass index; VISA-P, Victorian Institute of Sport Assessment-Patellar tendon; VAS, visual analog scale for pain; ^#^*n* = 15 because of two non-athletes in each group. Bias-corrected and accelerated (BCa) 95 percent bootstrap confidence interval.*

### Muscle Strength and Tendon Properties

Maximal knee extension torque and tendon morphological, mechanical, and material properties are reported in Table 2.

**TABLE 2:** Knee extension maximal torque and patellar tendon properties.

	Tendinopathy	Controls	*t*-Value_(16)_	Difference BCa 95% CI	*P*-Value	*d*-Value
Isometric torque (N⋅m)	204 ± 14	226 ± 19	−1.29	−22 [−57, 10]	0.216	−0.31
**Tendon dimensions**						
Tendon length (mm)	49.6 ± 1.7	46.7 ± 1.6	0.85	0.2 [−0.2, 0.6]	0.410	0.21
Tendon mean CSA (mm^2^)	148 ± 7.8***	111 ± 5.5	−4.50	37 [22, 52]	<0.001	−1.09
**Ramp contraction**						
Peak force (N)	5271 ± 287	5909 ± 534	−1.39	−638 [−1745, 302]	0.185	−0.34
Stress (MPa)	35.8 ± 1.4**	53.9 ± 4.7	−3.70	−18.1 [−28.5, −10.1]	0.002	−0.90
Strain (%)	8.8 ± 0.4	8.6 ± 0.4	0.49	0.3 [−0.9, 1.3]	0.634	0.12
Stiffness_max_ (N⋅mm^–1^)	2029 ± 170**	2617 ± 210	−3.07	−587 [−939, −234]	0.007	−0.74
Stiffness_com_ (N⋅mm^–1^)	1745 ± 146	1887 ± 75	−0.80	−142 [−430, 210]	0.435	−0.19
Young’s modulus_max_ (GPa)	0.69 ± 0.05**	1.10 ± 0.09	−3.88	−0.41 [−0.63, −0.23]	0.001	−0.94
Young’s modulus_com_ (GPa)	0.58 ± 0.04**	0.85 ± 0.06	−3.49	−0.27 [−0.43, −0.12]	0.003	−0.85
**Triangular loading ramps** (Figures 3A,B)						
Hysteresis (%)	18.6 ± 1.7	17.1 ± 1.0	0.76	1.4 [−1.9, 4.8]	0.461	0.18
Strain energy (J)	7.0 ± 0.5	8.5 ± 1.2	−1.48	−1.5 [−3.7, 0.3]	0.656	0.36

Values are expressed as mean ± standard error of the mean. Bias-corrected and accelerated (BCa) 95 percent bootstrap confidence interval. **p < 0.01, ***p < 0.001 compared with healthy control patellar tendons.

**FIGURE 1:**
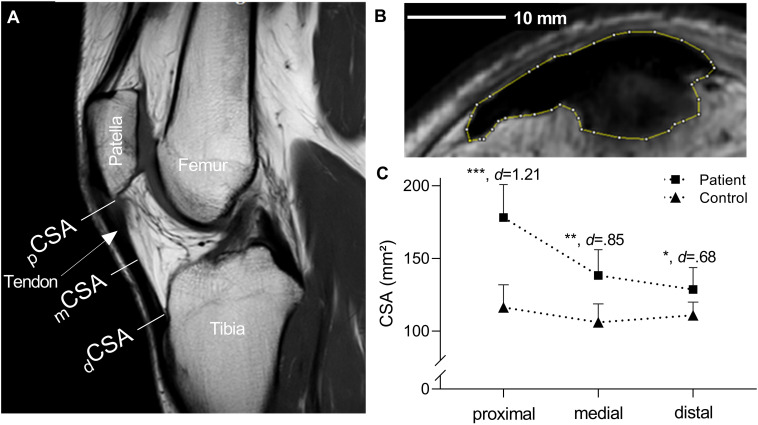
Regional patellar tendon cross-sectional areas (CSA). **(A)** Sagittal magnetic resonance (MRI) scan of the patellar tendon and denoted positions of CSA measurements. **(B)** Segmented, axial MRI of patellar tendon proximal-region in one representative patient with tendinopathy. **(C)** Between-group comparisons of patellar tendon CSA. Values are mean ± 95% confidence limits. _p_CSA, cross-sectional area proximal; _m_CSA, cross-sectional area medial; _d_CSA, cross-sectional area distal; *p < 0.05, **p < 0.01, ***p < 0.001 compared with healthy control patellar tendons.

Patellar tendon length was similar between groups, but mean CSA was significantly increased in the tendinopathic group. Region-specific comparison of CSA indicated that the pathology caused a significant tendon swellings at the proximal (+61%, BCa 95% [32.6, 86.4], *t*_(16)_ = 4.98, *p*< 0.001, *d* = 1.21), medial (+36%, BCa 95% [16.5, 48.7], *t*_(16)_ = 3.51, *p* = 0.003, *d* = 0.85), and distal tissue site (+17%, BCa 95% [5.9, 30.6], *t*_(16)_ = 2.80, *p* = 0.013, *d* = 0.68) (Figure 1).

**FIGURE 2:**
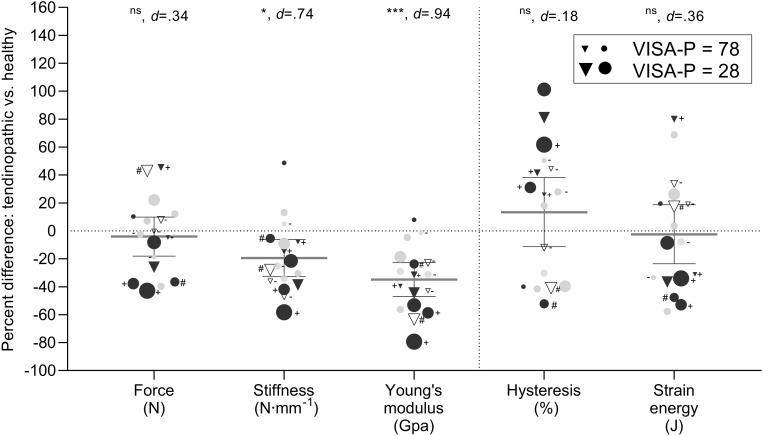
Percentage differences in tendon tensile force, mechanical, and material properties between chronic tendinopathic and healthy patellar tendon properties. Mechanical and material properties were evaluated at a common force level of each individual matched pair. Scatter plots with mean ± 95% confidence limits sizes as an approximate representation of clinical severity are shown (see figure legend). Circles indicate male and triangle female subjects. Black filled symbols indicate cyclic loading (long-distance running, football, American football), gray filled symbols spring-like loadings (volleyball, team handball), and white symbols skiers and recreational actives (no specific sport activity). The symbol label refers to vascularity and calcification, with no label means vascularization only, # calcification only, + both, and – nothing. ns, non-significant, *p < 0.05, ***p < 0.001 compared with intact patellar tendons.

**FIGURE 3:**
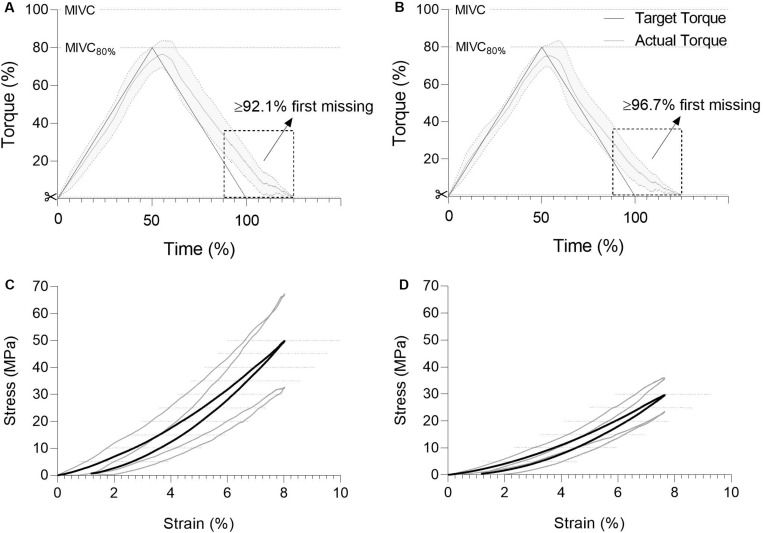
Between-group comparison of patellar tendon viscoelastic properties. Plots show the average torques via triangular loading ramp of the healthy **(A)**, and impaired subjects **(B)** plotted against time, and the associated mean stress-strain curves of the intact **(C)** and tendinopathic tendons **(D)**. MIVC, maximal isometric voluntary contraction. Values are means ± SD [gray area **(A,B)** and additional gray lines **(C,D)** of all subjects].

**FIGURE 4:**
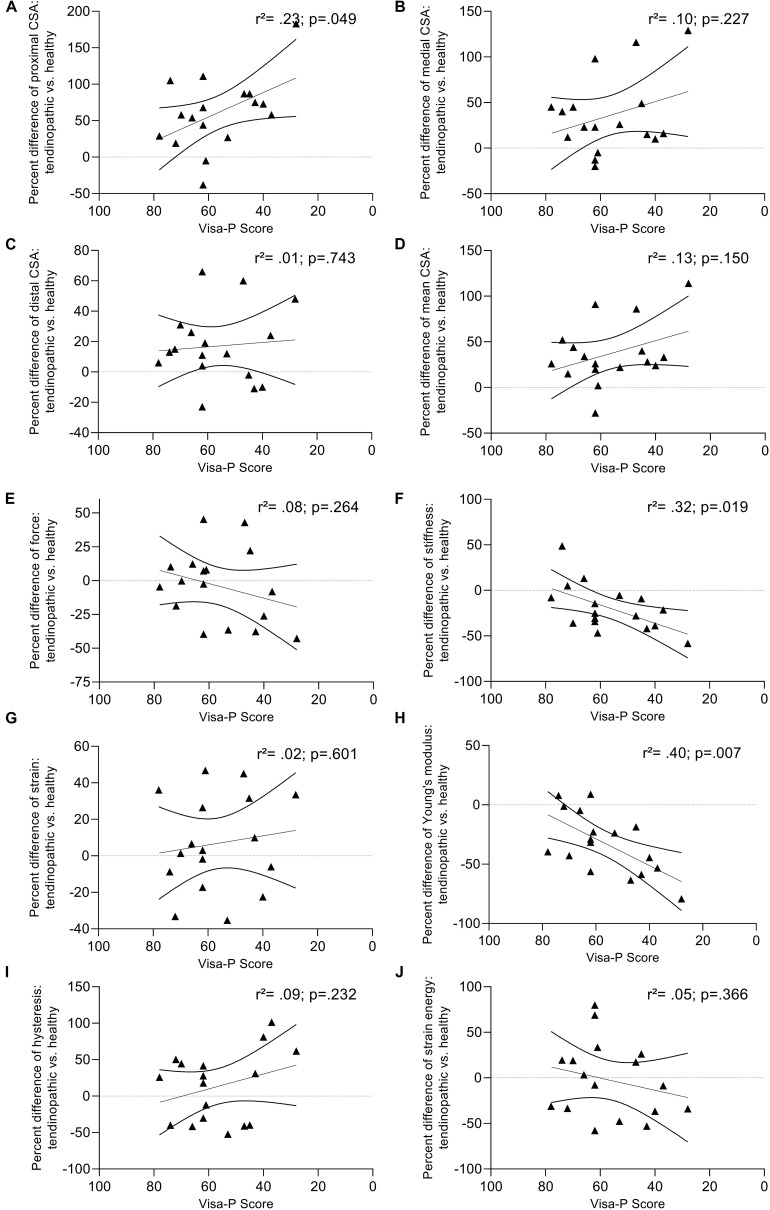
Differences in muscle strength and tendon properties of affected individuals and healthy controls plotted against the scores of the VISA-P questionnaire. Between-group comparison of patellar tendon dimension **(A–D)**, tendon tensile force **(E)**, stiffness **(F)**, strain **(G)**, Young’s modulus **(H)**, hysteresis **(I)**, and strain energy **(J)**. Lines depict the least-squares regression and their 95% confidence limits.

There was no difference in maximal knee extension torque or patellar tendon peak force when testing tendon mechanical properties. Patellar tendon stiffness did not differ between groups when measured at the greatest common force level. However, when measured at the individual highest force level, patellar tendon stiffness was lower (−18%) in the tendinopathic group compared to controls. Additionally, patellar tendon stress (−27%) and absolute and relative Young’s modulus (−32 and −33%) were significantly lower in tendinopathic patients compared to controls, but there was no difference in tendon strain (Table 2 and Figure 2). Patellar tendon hysteresis and elastic energy storage seem unaffected by tendinopathy (Table 2 and Figure 3). Furthermore, there was a significant correlation between the impaired patellar tendon properties and the clinical severity of symptoms represented by the scores of the VISA-P questionnaire for proximal tendon CSA, tissue stiffness and Young’s modulus (Figure 4).

## Discussion

Despite the substantial structural and histological changes occurring with tendinopathy (Magnusson et al., 2010) energy dissipation remained unchanged in the affected tendons included in this study. Additionally, differences in tendon stiffness between tendinopathic and healthy tendons were only seen at maximal individual force and not at common force level.

Patient reports of VISA-P and VAS scores (Table 1) are consistent with similar cross-comparison studies of individuals with chronically affected tendons (Couppé et al., 2013; Lee et al., 2017) and demonstrate the severe functional impairment of the included cohort. However, in contrast to a previous observation (Couppé et al., 2013; Helland et al., 2013) affected patellar tendons were larger near the affected region (CSA +61%), but also in tissue areas with no apparent hypoechoic areas, neovascularization or presence of worse-aligned collagen structure (Figure 1). We can neither elucidate mechanisms nor discern whether the observed tissue thickening is the cause or the consequence of the pathological processes, but these findings suggest that tendinopathy also affects tissue sites that appear asymptomatic on MRI and ultrasound images.

Consistent with previous observations, the greater tendon size was not reflected in higher stiffness values, but chronically diseased tendons exhibited a lower stiffness in high loading conditions (stiffness_max_) and a lower Young’s modulus. Contrasting differences in tissue size and stiffness between tendinopathic and healthy patellar tendons had been previously observed by Helland et al. (2013) (proximal CSA +19%; stiffness −20%), with a similar trend in the findings from Kongsgaard et al. (2010) (stiffness -9%). Other studies have, however, not found any difference between the tendon stiffness of affected and healthy control patellar tendons (Couppé et al., 2013) or even +22% higher values, though not statistically significant (Lee et al., 2017). Taken together, the different outcome obtained when measuring stiffness at individual or common maximal force level and these aforementioned studies suggest that part of the discrepancy is attributable to methodological factors. Previous studies assessing patellar tendon mechanical properties have typically measured stiffness at a submaximal force level, corresponding to the highest force common to all subjects (Kongsgaard et al., 2010; Couppé et al., 2013; Helland et al., 2013; Lee et al., 2017). While this standardizing approach is appropriate for homogenous sample populations, stiffness may be inadequately measured outside the linear portion of the force-deformation relationship in groups with broader variations in force (Seynnes et al., 2015). In our sample population, tendon force ranged from 3063 to 10,963 N, indicating that the stiffness of the stronger subjects was measured in the toe region. In contrast, another study found a lower stiffness in affected vs. healthy patellar tendons using both calculation methods (Helland et al., 2013). However, the force level common to all subjects in that study (∼70% of control max force) was substantially higher than in the current one (∼55% of control max force), supporting the idea of poor suitability of this approach for our data. Hence, although stiffness values may be influenced by the force level at which they were calculated (O’brien et al., 2010) the advantage conferred by the consideration of force levels closest to the elastic portion of the deformation curve seems to prevail over the methodological inconsistency described above. For this reason, we contend that patellar tendon stiffness is reduced with tendinopathy. Interestingly, however, the lower stiffness measured at maximal individual force in the symptomatic tendons was not reflected by any difference in tendon maximal strain. This observation is congruent with the findings of other studies (Helland et al., 2013) and may reflect the different effects of tendon structure and composition on tissue behavior at various levels of tensile load.

Conversely, the similar tissue hysteresis and energy storage capacity of both patients and controls suggest that pathological patellar tendons retain a similar ability to use elastic energy. High elastic modulus, large strains, and relatively low hysteresis (∼9%) are common features of various mammalian tendons (Ker, 1981; Bennett et al., 1986; Shadwick, 1990; Matson et al., 2012) and make tendons the essential source of elastic storage and recovery during locomotion (Roberts, 2016). The hysteresis values (∼17–18%) measured here were much higher in comparison to the *in vitro* studies mentioned above. Aside from differences due to methodology, such a high hysteresis may indicate a lower energy-conserving capacity and a greater heat accumulation during prolonged exercise. Nonetheless, the findings are in accordance with previous *in vivo* measurements (Finni et al., 2013 for review). Regardless of the magnitudes measured here, the results of this study suggest that tendinopathy does not affect the hysteresis and thus the performance of the myotendinous complex during locomotion or risk of further injury via intra-tendinous hyperthermia. However, this finding contrasts with observations of higher hysteresis, lower storage and release of elastic energy and performance deficits in elite athletes with mid-portion Achilles tendinopathy (Wang et al., 2012) and could suggest that tendinopathy affects the mechanical properties of patellar and Achilles tendons differently. Such tendon-specific discrepancies are in line with the distinct functional (Kurokawa et al., 2001), metabolic (Kubo and Ikebukuro, 2012), and anatomical (Basso et al., 2001; Szaro et al., 2009) features reported previously. Moreover, these observations highlight that advanced stage tendinopathy may affect the various mechanisms driving tensile loading and unloading of the patellar tendon differently.

The significant correlations found between VISA-P scores and the differences in proximal CSA, stiffness and Young’s modulus (Figure 4) of affected individuals and healthy controls reinforce the differences found in these variables and suggest that the clinical severity is reflected in tendon properties. The discrepancy between studies measuring the mechanical properties of symptomatic tendons could, therefore, be partly explained by differences in symptom severity as measured with this scale. However, the lack of association between VISA-P and the differences in other objective measures of tendon properties (e.g., capacity to store and release elastic energy) indicates the complexity of this multifactorial pathology. Thus, symptoms typically associated with tendinopathy are not necessarily linked to patient perception nor to mechanical changes in the tendon. The present data do not support the influence of other outcome measures such as the influence of sex, loading pattern, or the occurrence of clinical signs such as neovascularization and/or calcification on tendon properties. These correlation analyses are, however, based on case-control pairs and should be considered with caution. Additional studies with larger cohorts and/or a within-subject design are needed to confirm the observed associations.

## Conclusion

The comparison of case-control pairs of symptomatic and pain-free, visually asymptomatic patellar tendons confirmed morphological differences and indicated differences in certain mechanical and material properties. Tendinophatic patellar tendons were characterized by a much larger thickness but a lower stiffness (and consequently a lower Young’s modulus) at maximal individual force. On the other hand, analyses of the loading and unloading curves suggest that elastic energy storage and dissipation capacities remain similar in tendinopathic and healthy subjects. Besides, the significant correlations found between the functional severity of the pathology (VISA-P) and mechanical and material patellar tendon properties (Figure 4) calls for caution when interpreting findings on a group mean level only. The severity of the pathology also affects patellar tendon mechanical properties. Future studies are needed to evaluate if these differences in tendon properties can be used as a predictor of pathology occurrence and whether they are reversible. In addition, upcoming studies should also assess the possible impact of the lower patellar tendon stiffness on muscular function in sporting or daily activities.

### Limitations

Potential limitations should be considered before concluding. First, some of the subjects suffered from patellar tendon disorders substantially longer than others (Table 1). Although symptom duration reportedly affects tissue cells and extracellular matrix differently (Dakin et al., 2015) a symptom duration difference within the present range of 10–120 months is unlikely to have influenced our results. This assumption is supported by the findings of the present study, with a lack of associations between symptom duration and both function and tendon property differences. Interpretations should, however, be considered in relation to the included cohort with sport-specific high volume (e.g., long-distance running) and high intensity (e.g., volleyball) tissue loading. Second, tensile forces on the patellar tendon are demonstrably influenced by the simultaneous activation of the antagonist’s muscles (Maganaris, 2001). However, muscle strength cannot be measured directly *in vivo*, and the quantitative nature of the EMG-strength relationship remains a topic of scientific debate. Despite these potential validation limits, it is unlikely that differences in coactivation have influenced our findings. Similar ratios of antagonist-to-measured knee extension torque in tendinopathic (11.2%) and healthy subjects (10.1%) support this assumption. Finally, tendon provides viscoelastic behavior, and tissue property acquisitions potentially differ during dynamic, real-life situations (Kösters et al., 2014). However, the methodology used in the present study is currently the only way to estimate tendon properties validly and reliably *in vivo*. Moreover, it seems a fair assumption that tendon properties calculated during isometric ramp contractions would be related to their measurements under different contractile conditions. Therefore, this standardized procedure seems valid and does not mitigate our initial conclusions.

## Data Availability Statement

The datasets generated for this study are available on request to the corresponding author.

## Ethics Statement

The studies involving human participants were reviewed and approved by the German register for clinical trials: DRKS00011338 Local Research Ethics Committee: 415-E/2012/11-2016. The patients/participants provided their written informed consent to participate in this study.

## Author Contributions

H-PW, OS, AK, EM, and FR contributed to the conceptualization of the study, and the review and editing of the writing. H-PW contributed to the methodology, the writing – original draft visualization, and the supervision. H-PW and FR contributed to the investigation and the project administration. H-PW contributed to the data analysis. FR, H-PW, and OS contributed to the funding acquisition. All authors read and approved the content of the final manuscript.

## Conflict of Interest

The authors declare that the research was conducted in the absence of any commercial or financial relationships that could be construed as a potential conflict of interest.

## Footnotes

1 http://imagej.nih.gov/ij/
