# Supplementary material for: Mechanical and Material Tendon Properties in Patients With Proximal Patellar Tendinopathy
Source: Front Physiol. 2020 Jun 24;11:704. doi: 10.3389/fphys.2020.00704 (PMC7358637; doi:10.3389/fphys.2020.00704)
Supplement: Supplementary file 1 [file Table_1.DOCX]

|  | **Assessed for eligibility** | |  |
| --- | --- | --- | --- |
|  | Tendinopathy (n=52) | Healthy (n=34) |  |
|  |  |  |  |
| \| Exclucded (n=35)   - declined to participate (n=2) - not meeting inclusion criteria (n=25) - VISA-P > 80 (n=9) and/or - not proximal (n=5) - no hypoechoic area (n=2) - no tissue thickening (<1mm; n=7) - pre-injury or surgery of the knee (n=3) - Osgood-Schlatter disease (n=4) - Tendon treatments (e.g., drug abuse,   injection of substances…) (n=5)   - No match to healthy control (n=8) \| \| --- \| |  |  | Excluded (n=17)   - not meeting inclusion criteria (n=14) - symptomatic images (n=7) - pre-injury or surgery of the knee (n=3) - Osgood-Schlatter disease (n=3) - shoulder implant (n=1) - worse match (n=3) |
|  |  |  |  |
|  | **Allocation**  (n=34) | |  |
|  |  |  |  |
| Tendinopathy group (n=17) | ***Matching criteria*** | | Control group (n=17) |
| *same sport (~same experience yrs)*  *(football (n=12),volleyball (n=8), team handball (n=4), skiing (n=4), long distance running (n=2), American football (n=2),recreational active (n=2))*  *same level (national or international)*  *same sex*  *~same anthropometrics (age, height, body mass)* | | | |

**Figure 1: Flow chart of the study population selection process and the inclusion and exclusion criteria.**
